# Multiple facets of HIV-associated renal disease

**DOI:** 10.1590/1414-431X20165176

**Published:** 2016-03-18

**Authors:** D.R. da Silva, I.C. Gluz, J. Kurz, G.G. Thomé, R. Zancan, R.N. Bringhenti, P.G. Schaefer, M. dos Santos, E.J.G. Barros, F.V. Veronese

**Affiliations:** 1 Serviço de Nefrologia, Hospital de Clínicas de Porto Alegre, Porto Alegre, RS, Brasil; 2 Serviço de Patologia, Hospital de Clínicas de Porto Alegre, Porto Alegre, RS, Brasil

**Keywords:** HIV, Renal disease, Proteinuria, Collapsing focal segmental glomerulosclerosis, CD4 cell count, Chronic kidney disease

## Abstract

HIV infection has a broad spectrum of renal manifestations. This study examined the clinical and histological manifestations of HIV-associated renal disease, and predictors of renal outcomes. Sixty-one (64% male, mean age 45 years) HIV patients were retrospectively evaluated. Clinical presentation and renal histopathology were assessed, as well as CD4 T-cell count and viral load. The predictive value of histological lesion, baseline CD4 cell count and viral load for end-stage renal disease (ESRD) or death were determined using the Cox regression model. The outcomes of chronic kidney disease (CKD) and ESRD or death were evaluated by baseline CD4 cell count. The percent distribution at initial clinical presentation was non-nephrotic proteinuria (54%), acute kidney injury (28%), nephrotic syndrome (23%), and chronic kidney disease (22%). Focal segmental glomerulosclerosis (28%), mainly the collapsing form (HIVAN), acute interstitial nephritis (AIN) (26%), and immune complex-mediated glomerulonephritis (ICGN) (25%) were the predominant renal histology. Baseline CD4 cell count ≥200 cells/mm^3^ was a protective factor against CKD (hazard ratio=0.997; 95%CI=0.994-0.999; P=0.012). At last follow-up, 64% of patients with baseline CD4 ≥200 cells/mm^3^ had eGFR >60 mL·min^-1^·(1.73 m^2^)^-1^ compared to the other 35% of patients who presented with CD4 <200 cells/mm^3^ (log rank=9.043, P=0.003). In conclusion, the main histological lesion of HIV-associated renal disease was HIVAN, followed by AIN and ICGN. These findings reinforce the need to biopsy HIV patients with kidney impairment and/or proteinuria. Baseline CD4 cell count ≥200 cells/mm^3^ was associated with better renal function after 2 years of follow-up.

## Introduction

In 2014, the Brazilian Ministry of Health estimated there were 734,000 people living with HIV/AIDS in Brazil, corresponding to a prevalence of 0.4% (1). HIV-associated renal involvement was first described in 1984 (2 3 4), and since then several forms of HIV-associated nephropathy have been described (5 6 7 8 9 10 11 12 13 14) resulting from the direct effects of virus infection on the kidneys or indirect effects of intercurrent illness or medications.

In the 80's, the clinical spectrum of HIV-associated renal disease was described as nephrotic and nephritic syndrome, acute kidney injury (AKI), chronic kidney disease (CKD), and non-nephrotic proteinuria (14). The classical HIV-associated nephropathy (HIVAN) is known to occur more frequently among HIV-infected individuals of African descent, and its clinical manifestations include nephrotic proteinuria, hematuria, rapidly progressive renal failure, and hypertension. HIVAN is characterized histologically by a collapsing focal segmental glomerulosclerosis (FSGS) (2,6,10,14). A non-collapsing FSGS and immune complex-mediated glomerulonephritis (ICGN) have been described, such as membranoproliferative glomerulonephritis, mesangial proliferative glomerulonephritis, membranous nephropathy, IgA nephropathy and lupus-like glomerulonephritis (5,) as well as thrombotic microangiopathy (8,9). Other HIV-associated conditions, including bacterial or viral infections and nephrotoxicity of antiretroviral therapy (ART), induce specific kidney damage such as acute tubular necrosis (ATN) and acute interstitial nephritis (AIN) (8,10). Lastly, kidney injury caused by amyloidosis, diabetes mellitus, and hypertension are also part of the spectrum of HIV-associated renal diseases (8) because they are comorbidities that nowadays develop owing to the increased survival of these patients. For example, CKD is currently considered an independent risk factor for mortality in HIV patients (15 [Bibr B16]).

Renal biopsy is indicated in HIV patients when they present significant signs of kidney damage for accurate diagnosis of the underlying cause, appropriate treatment, and prognosis (8 10,15,). Treating patients with HIVAN using ART, corticosteroids, cyclosporin and/or angiotensin inhibitors may reduce proteinuria and preserve kidney function as reported by Elewa et al. (11) and Kalayjian (17) in their review studies. However, the benefits of ART and other drug treatments on non-HIVAN lesions are not yet clear (6,10,11,17). Undoubtedly, the treatment of different co-morbidities that develop in aging HIV patients, such as hypertension, diabetes, and non-AIDS diseases is of paramount importance for their longer survival.

This study aimed to describe the findings of renal biopsies in HIV patients, correlating histopathological results and clinical presentation, and to search for predictors of CKD progression and death.

## Material and Methods

### Patient selection

This retrospective cohort study included 61 HIV patients (64% male, mean age 45 years) who had a kidney biopsy done for acute, acute-on-chronic or chronic kidney failure of unknown etiology, pathological proteinuria with or without hematuria, nephrotic syndrome or nephritic syndrome. These patients were attended at a teaching hospital in Southern Brazil, and were selected throughout the period of January 2004 to December 2014. Patients on chronic dialysis treatment and kidney transplant recipients were excluded, as well as those with incomplete medical records. This study was approved by the Research Ethics Committee of Hospital de Clínicas de Porto Alegre.

### Demographic characteristics and clinical data

The patients' demographic characteristics, clinical presentation, comorbidities, presence of viral co-infections and active infections of any origin at the time of renal biopsy were collected. Renal biopsy specimens were routinely examined by two nephropathologists from our institution. HIVAN was defined by the presence of collapsing glomerular lesions, podocyte proliferation, microcystic tubular dilatation and flattening and atrophy of tubular cells (18). The histological diagnosis of FSGS in the absence of these features was defined as non-collapsing FSGS. The following laboratory data were collected: serum creatinine (automated Jaffe method, Modular Analytics, Roche Diagnostics, Germany); estimated glomerular filtration rate (eGFR) using the CKD-EPI formula; and serum albumin and proteinuria determined by urine protein-to-creatinine ratio (Pr/Cr) in a random urine sample. Chronic kidney disease stage was established according to Kidney Disease Improving Global Outcomes classification (19) for the CKD patients. CD4 cell count (cells/mm^3^) was measured by flow cytometry (BD FACSCalibur flow cytometer, BD Biosciences, USA) and the quantification of viral load (HIV-1 RNA copies/mL) was performed by real-time polymerase chain reaction (RT-PCR; Abbott m2000sp extraction system, Abbott Laboratories, USA), both at the time of renal biopsy and at last follow-up.

### Statistical analysis

Data are reported as absolute frequencies and percentages, means±SD or medians and interquartile ranges. The association between categorical variables was assessed using the chi-square test or Fisher's exact test. The paired*t*-test, the independent samples *t*-test, Wilcoxon and Mann-Whitney tests were performed as appropriate for comparisons between two groups. The predictive values of baseline serum creatinine, histological diagnosis, baseline CD4 cell count and logarithm of the viral load (log viral load) for the outcome end-stage renal disease (ESRD) or death were determined using the Cox regression model. Survival curves for the outcomes eGFR <60 mL·min^-1^·(1.73 m^2^)^-1^ and ESRD or death by baseline CD4 cell count (≥200 and <200 cells/mm^3^) were analyzed by the Kaplan-Meier method. All analyses were performed using SPSS for Windows (version 18.0, SPSS Inc., USA). The level of significance was set at P<0.05.

## Results

Sixty-one HIV patients with clinical indication for renal biopsy were selected. The patients' clinical and demographic characteristics are shown in Table 1. Most were young, white male patients. One-third had hypertension and 16% diabetes mellitus. Fifteen patients were co-infected with hepatitis C virus (25%) and 3 with hepatitis B virus (5%). Of those diagnosed with HIVAN, 5 (29%) were co-infected with hepatitis C virus and 1 (6%) with hepatitis B virus. Most patients (82%) were on ART. The ART regimen at kidney biopsy included: tenofovir (36%), efavirenz (29%), lamivudine (68%), zidovudine (25%), ritonavir (26%), atazanavir (21%), lopinavir (19%), abacavir (16%), and nevirapine (9%). Thirty-five (58%) patients were taking an angiotensin-converting enzyme inhibitor (iECA) or an angiotensin II receptor blocker (ARB). Follow-up time after renal biopsy ranged from 9 to 46 months (median of 25 months).

**Table:**
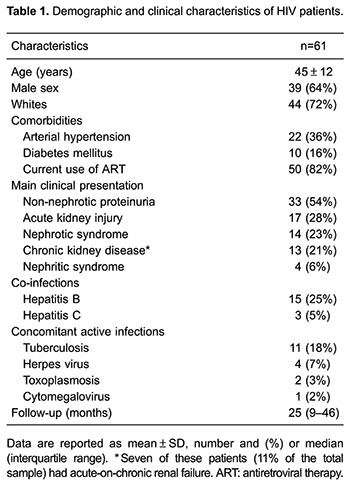

Active infections at the time of renal biopsy included tuberculosis (18%), herpes virus infection (7%), toxoplasmosis (3%), and cytomegalovirus (2%). The clinical presentations of the renal disease at study entry were non-nephrotic proteinuria (54%), acute kidney injury (28%), nephrotic syndrome (23%), and CKD (21%, 13 patients). Of patients with CKD, 7 (11% of the total sample) had acute-on-chronic renal failure. Four (6.5%) patients presented with nephritic syndrome. The CKD stage of the 13 patients prior to entry into the study was: 2 (15%) in stage 2, 3 (23%) in stage 3a, 3 (23%) in stage 3b, and 5 (39%) in stage 4.

**Figure 1:**
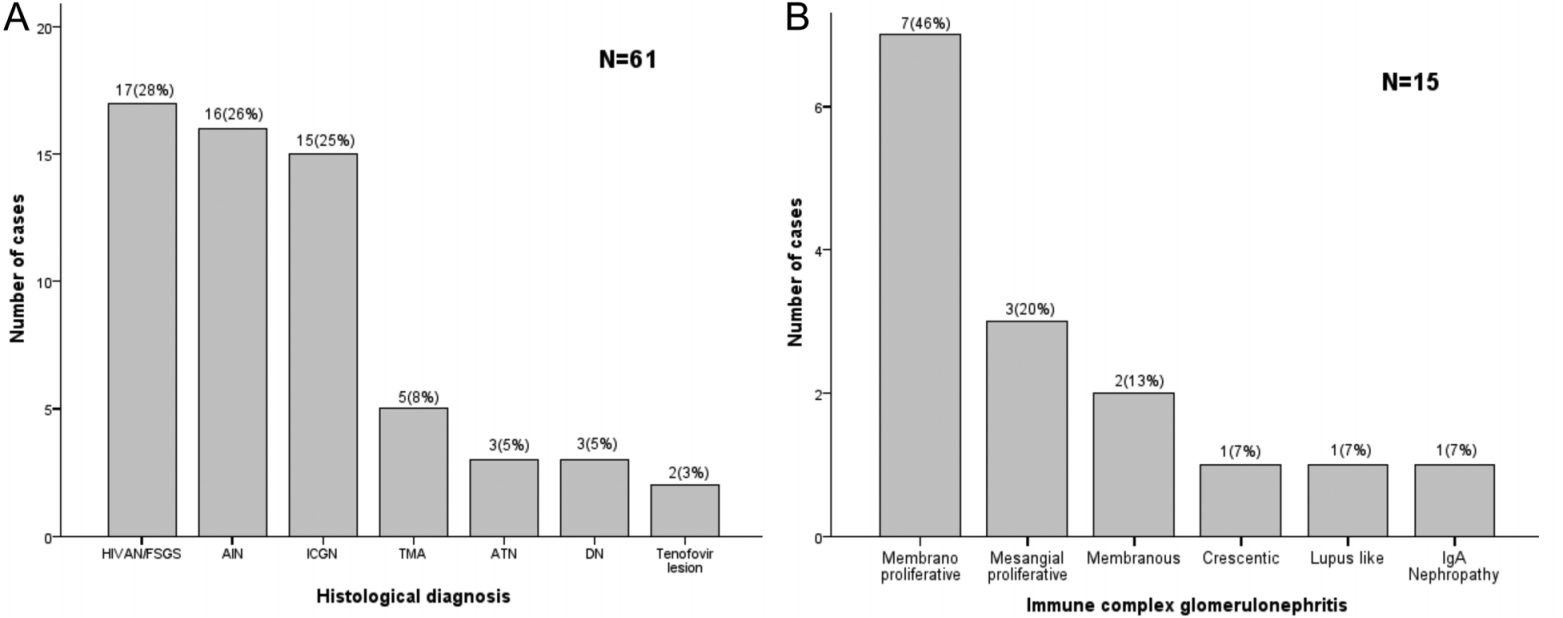
Histological diagnosis of kidney biopsies. *Panel A*, Prevalence of histological lesions in HIV patients. Tenofovir lesion refers to acute kidney injury and acute tubular necrosis related to tenofovir nephrotoxicity. HIVAN/FSGS: HIV-associated nephropathy/non-collapsing focal segmental glomeruloscleriosis; AIN: acute interstitial nephritis; ICGN: immune complex-mediated glomerulonephritis; TMA: thrombotic microangiopathy; ATN: acute tubular necrosis; DN: diabetic nephropathy. *Panel B*, Prevalence of histological types of immune complex-mediated glomerulonephritis.

A variety of histological lesions was found in renal biopsy specimens (Figure 1A). The main histological diagnoses were FSGS (28%; 14 HIVAN and three non-collapsing FSGS); AIN (26%), and ICGN (25%). AKI caused by tenofovir was presumed in 2 patients, because they recovered renal function after drug withdrawal. Three patients with diabetes showed histological findings consistent with diabetic nodular glomerulosclerosis, and 5 patients thrombotic microangiopathy (TMA). Of 15 patients with ICGN (Figure 1B), membranoproliferative glomerulonephritis was seen in 7 and mesangial glomerulonephritis in 3. Regarding the association between clinical presentation and histological diagnosis, HIVAN or non-collapsing FSGS and ICGN were predominantly manifested as non-nephrotic proteinuria or nephrotic syndrome; AIN and ATN as acute kidney injury; HIVAN or non-collapsing FSGS manifested also as CKD, as well as TMA and diabetic nephropathy.

A comparison between patients with HIVAN or non-collapsing FSGS (n=17, 28%) and the other patients (n=44, 72%) revealed in the former group a greater proportion of African descent (48 *vs* 19%; P=0.041), nephrotic proteinuria (53*vs* 14%; P=0.033), and more patients with worse outcomes, such as eGFR <60 mL·min^-1^·(1.73 m^2^)^-1^ (88*vs* 40%; P=0.006) and ESRD or death (65 *vs* 32%; P=0.021). The percentage of interstitial fibrosis and tubular atrophy identified in renal biopsy specimens was on average higher in patients with HIVAN or non-collapsing FSGS (24±18%) compared with those with other histological types (10±17%; P=0.041). However, there was no difference in CD4 cell counts between these two groups (199 [118-491] *vs* 220 [87-435 cells/mm^3^], P=0.623, respectively). As per drug protocol, all patients with HIVAN or non-collapsing FSGS were on ART, compared to 72% in the other group (P=0.130).

Table 2 shows laboratory results at the time of renal biopsy and at the end of follow-up. Compared to baseline values there was a significant reduction in Pr/Cr (P=0.003) and a significant increase in eGFR (P=0.005) and serum albumin (P=0.041). CD4 cell counts increased and viral load decreased in the last assessment, although these changes were not statistically significant (P=0.170 and P=0.390, respectively).

**Table:**
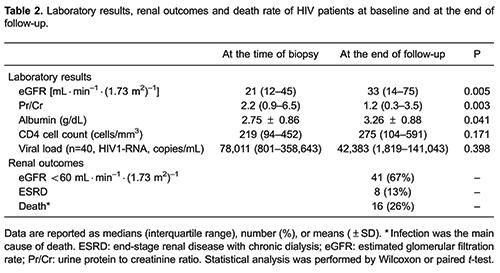

At the end of follow-up (median of 25 months), 41 (67%) patients had eGFR below 60 mL·min^-1^·(1.73 m^2^)^-1^ (Table 2). These patients did not differ from those with eGFR ≥60 mL·min^-1^·(1.73 m^2^)^-1^ in age, initial serum creatinine, proteinuria, histological diagnosis, and time of follow-up, but a greater proportion had CD4 cell count <200 cells/mm^3^: 23 of 41 (56%)*vs* 5 of 20 (23%), respectively (P=0.011). Of the 30 patients with renal failure at study entry (acute, n=17; acute-on-chronic, n=7; or CKD, n=6), 2 diagnosed with acute kidney injury, 3 diagnosed with acute-on-chronic kidney disease and 3 diagnosed with CKD (8 patients, 13%) progressed to ESRD requiring chronic hemodialysis. Sixteen of the 24 patients with acute renal failure recovered renal function partially or completely. Sixteen (26%) of the patients died, and the main cause of death was infection.

Thirty-five patients were taking iECA or ARB at the time of kidney biopsy. This group showed higher levels of Pr/Cr than the patients not using these drugs [3.0 (2.0-7.5)*vs* 0.9 (0.3-2.7), P<0.001, respectively] at study entry, but not at the end of follow-up [1.7 (0.2-4.9) *vs* 0.7 (0.3-3.3), P=0.216, respectively]. In addition, there was a trend to higher eGFR at study entry [23 (15-46) *vs* 15 (9-35 mL·min^-1^·(1.73 m^2^)^-1^), P=0.081, respectively], but no difference was found at the end of follow-up [31 (17-75) *vs* 33 (14-78 mL·min^-1^·(1.73 m^2^)^-1^), P=0.641, respectively]. End-stage renal disease and death rates were similar between patients using or not using iECA or ARB: 13.8 *vs* 15% (P=0.475) and 41 *vs*25% (P=0.210), respectively, even when considering these two outcomes together (55*vs* 40%, P=0.381, respectively).

**Figure 2:**
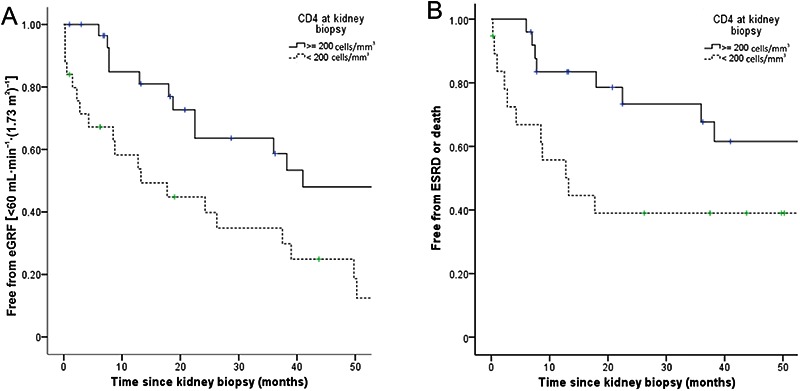
Renal outcomes according to baseline CD4 cell count. *Panel A*, proportion of patients free from chronic kidney disease [defined as eGFR <60 mL·min^-1^·(1.73 m^2^)^-1^] during follow-up among those with CD4 cell count ≥200 cells/mm^3^ compared to those with CD4 cell count <200 cells/mm^3^ at biopsy (P=0.003); eGFR: estimated glomerular filtration rate. *Panel B*, proportion of patients free from end-stage renal disease (ESRD) or death during follow-up among those with CD4 cell count ≥200 cells/mm^3^ compared to those with CD4 cell count <200 cells/mm^3^ at biopsy (P=0.027, Cox regression).

The Kaplan-Meier curves (Figure 2A) showed that patients with baseline CD4 cell count ≥200 cells/mm^3^ compared to those with CD4 cell count <200 cells/mm^3^ had more preserved kidney function over a median follow-up of 25 months (64 *vs* 35% without eGFR <60 mL·min^-1^·(1.73 m^2^)^-1^; log rank=9.043, P=0.003). The same was seen for ESRD or death (log rank=4.878, P=0.027) (Figure 2B). Confirming these results, the Cox regression analysis revealed that baseline CD4 cell count ≥200 cells/mm^3^ was a protective factor against ESRD or death (P=0.012). There was a trend toward an increase in log viral load in the risk for this outcome (P=0.08). Baseline serum creatinine and histological lesion in renal biopsy were not predictors of CKD progression, dialysis or death.

## Discussion

This study found a broad clinical spectrum of HIV-associated renal diseases among patients presenting AKI, proteinuria, and/or CKD. As histological diagnosis, HIVAN, AIN, and ICGN were prevalent. Baseline CD4 cell count was found to be a predictor of CKD and ESRD or death.

With the widespread use of ART in the last 15 years, the spectrum of HIV-associated renal diseases has significantly changed to include not only HIVAN but also non-collapsing FSGS, AIN, ICGN, diabetic nephropathy and amyloidosis among others (12,20). The clinical course of these conditions is characterized by slow and progressive loss of kidney function with low levels of proteinuria (21). Berliner et al. (10) studied 152 HIV patients who underwent renal biopsy between 1995 and 2004 and identified HIVAN in 35%, non-collapsing FSGS in 22%, and AIN in 7.9%. In this study, CD4 cell count ≥200 cells/mm^3^ and preserved GFR were predictors of non-collapsing FSGS while HIVAN was associated with ESRD and reduced patient survival.

Szczech et al. (6) investigated 87 HIV patients in six different study sites in the USA and found HIVAN in 47%; only 27 (31%) patients were on ART. The difference in the prevalence of ART use found in our study (82%) compared with that reported by Szczech et al. 12 years earlier may be explained by the availability of treatments and a wider variety of antiretroviral regimens now available, which possibly determined a lower prevalence of HIVAN in our sample. Since the late 1990s, ART has successfully controlled viral replication improving immune function, patient survival and resulting in a more indolent course of HIV-associated renal diseases (11,21,22).

This hypothesis is further supported by the findings of Wearne et al. (13). They studied 192 South African HIV patients, where 79% showed histological signs of HIVAN; 57% had isolated HIVAN and 22% had HIVAN associated with ICGN on renal biopsy. The mortality rate was reduced by 57% in patients with HIVAN who had access to ART. The predictors of higher mortality rate in this study included low eGFR, increased proteinuria, and the presence of microcysts in renal biopsy. In contrast to our study, they found no correlation of CD4 cell counts with mortality. These authors also reported that ART in patients with HIVAN plus ICGN reduced proteinuria and slowed the progression of kidney function loss but did not affect patient survival. Although Wearne's study is the largest one published on HIV-associated renal diseases, one must bear in mind the potential effects of confounding factors, including low life expectancy of South African population, poor access to ART, and high prevalence of HIVAN known to be associated with treatment resistance and unfavorable renal outcomes.

It has been recently reported an increased risk of HIVAN in African-American (23) and South African (24) individuals with apolipoprotein L1 (APOL1) gene polymorphisms on chromosome 22. Having two APOL1 risk alleles increases by 50% the chance of developing HIVAN for untreated HIV-infected individuals at an earlier age and at risk of faster progression to ESRD. Moreover, HIV-1 subtypes are unevenly distributed in different geographical locations with diverse clinical implications: subtype B is predominant in Latin America (68-74%) and the more virulent subtype C, that can produce high viral counts, is the dominant form in Southern Africa (98%) (25 [Bibr B26]).

A study conducted in France (22) reported a prevalence of HIVAN of 29.5% in 88 patients with baseline CD4 cell count of 217 cells/mm^3^ at the time of biopsy; 26.1% had non-collapsing FSGS and 22.7% ICGN. The risk of HIVAN was found to be 16.8 times higher in individuals of African descent, 6.4 times higher with CD4 cell count <200 cells/mm^3^, and 21.4 times higher with eGFR <30 mL·min^-1^·(1.73 m^2^)^-1^. Our results are consistent with their findings, showing impaired kidney function among patients with HIVAN.

In our sample, 22% of patients presented predominantly membranoproliferative ICGN. Two of these patients had hepatitis C infection but without any evidence of cryoglobulinemia or active infection (undetectable viral load), which makes it more likely to be etiologically associated with HIV infection. These findings are corroborated by other studies describing a spectrum of diseases ranging from membranoproliferative glomerulonephritis, membranous glomerulonephritis, and IgA and non-IgA mesangial proliferative glomerulonephritis among others (5,7 9,21,). Non-HIVAN glomerulopathies account for an estimated one-third to half of kidney diseases in HIV-infected individuals (6). The risk factors and clinical course of these conditions are not yet clear; their treatment has been little investigated and its effects are uncertain (6,11,17).

Acute interstitial nephritis was the second cause of HIV-related renal disease in our sample. In the study by Szczech et al. (6), only 3 out of 89 HIV patients had interstitial nephritis. Potential etiologies were not reported. Parkhie et al. (27) found AIN in 11% of 262 biopsies of patients with HIV, and nonsteroidal anti-inflammatory drugs and sulfamethoxazole/trimethoprim use were identified as the most frequent causes; ARTs were related in three of those cases. Recently, atazanavir, one of the key drugs in combination ART, was associated with acute tubulointerstitial nephritis manifested as AKI or with slowly progressive CKD, in which a chronic tubulointerstitial nephritis with precipitation of atazanavir crystals is found in renal biopsy (28). Other authors also reported AIN as a cause of non-HIVAN renal disease in HIV patients undergoing biopsy (8,10).

Our study has limitations. The patients in our sample had clinical indication for renal biopsy, which may have caused an etiological diagnosis. However, HIV-associated renal disease may have an even larger spectrum, affecting individuals with few or no clinical and laboratory manifestations, which can underestimate disease burden and treatment. As a result, HIV patients may develop unfavorable outcomes with deteriorating kidney function because they do not benefit from early treatment and therefore progress to kidney function loss, ESRD, or death (29). Despite the widespread use of ART, HIV infection directly or indirectly causes kidney damage and the clinical suspicion of renal injury requires a kidney biopsy for confirmation. A liberal approach in renal biopsy indication for HIV individuals may lead to a shift in the clinical course of the disease. According to biopsy results, treatments may be redirected to highly active ART, corticosteroids and immunosuppressive drugs, plasmapheresis, angiotensin inhibitors, or even discontinuation of nephrotoxic drugs regardless of kidney function, CD4 cell counts and viral load (30). Overall, a tendency for better clinical outcomes in the era of modern ART has been recently demonstrated (31), as both the incidence of ESRD due to HIVAN and the mortality of HIV patients have decreased substantially.

In conclusion, histological studies together with clinical and laboratory features are crucial for prognostic assessment and treatment planning of HIV-associated renal diseases, focused on early ART and inhibition of the renin-angiotensin system. Individuals with HIVAN, ICGN or AIN should undergo careful evaluation and may benefit from tailored treatment approaches including corticosteroids and immunosuppressive drugs. The early diagnosis and specific treatment of HIV-associated renal diseases may prevent CKD progression and reduce mortality, a hypothesis to be tested in large prospective trials.
